# Analysis of factors associated with hiccups based on the Japanese Adverse Drug Event Report database

**DOI:** 10.1371/journal.pone.0172057

**Published:** 2017-02-14

**Authors:** Ryuichiro Hosoya, Yoshihiro Uesawa, Reiko Ishii-Nozawa, Hajime Kagaya

**Affiliations:** 1 Department of Clinical Pharmaceutics, Meiji Pharmaceutical University, Kiyose, Tokyo, Japan; 2 Department of Pharmacy, Japanese Red Cross Musashino Hospital, Musashino, Tokyo, Japan; National Chiao Tung University College of Biological Science and Technology, TAIWAN

## Abstract

Hiccups are occasionally experienced by most individuals. Although hiccups are not life-threatening, they may lead to a decline in quality of life. Previous studies showed that hiccups may occur as an adverse effect of certain medicines during chemotherapy. Furthermore, a male dominance in hiccups has been reported. However, due to the limited number of studies conducted on this phenomenon, debate still surrounds the few factors influencing hiccups. The present study aimed to investigate the influence of medicines and patient characteristics on hiccups using a large-sized adverse drug event report database and, specifically, the Japanese Adverse Drug Event Report (JADER) database. Cases of adverse effects associated with medications were extracted from JADER, and Fisher’s exact test was performed to assess the presence or absence of hiccups for each medication. In a multivariate analysis, we conducted a multiple logistic regression analysis using medication and patient characteristic variables exhibiting significance. We also examined the role of dexamethasone in inducing hiccups during chemotherapy. Medicines associated with hiccups included dexamethasone, levofolinate, fluorouracil, oxaliplatin, carboplatin, and irinotecan. Patient characteristics associated with hiccups included a male gender and greater height. The combination of anti-cancer agent and dexamethasone use was noted in more than 95% of patients in the dexamethasone-use group. Hiccups also occurred in patients in the anti-cancer agent-use group who did not use dexamethasone. Most of the medications that induce hiccups are used in chemotherapy. The results of the present study suggest that it is possible to predict a high risk of hiccups using patient characteristics. We confirmed that dexamethasone was the drug that has the strongest influence on the induction of hiccups. However, the influence of anti-cancer agents on the induction of hiccups cannot be denied. We consider the results of the present study to be helpful for the prevention and treatment of hiccups.

## Introduction

Hiccups are occasionally experienced by most individuals and are mainly caused by diaphragmatic myoclonus [1]. Myoclonus is the brief, involuntary twitching of a muscle or group of muscles. Hiccups are a type of myoclonic jerk that specifically affects the diaphragm. Although it is rare for hiccups to be life-threatening, they often reduce quality of life. The control of these symptoms is particularly important clinically because treatments may be disturbed when hiccups occur as an adverse effect.

A hiccup is an involuntary, spasmodic contraction of the diaphragm causing a beginning inspiration that is suddenly checked by closure of the glottis. The glossopharyngeal nerve (ninth cranial nerve), vagus nerve (tenth cranial nerve), nuclei of the solitary tract, nucleus ambiguus, and phrenic nerve are all involved in the afferent and efferent pathways of the hiccup reflex arc [1–4]. However, the exact mechanisms underlying the central link of the hiccup reflex arc are not clear. There are three hiccup classifications based on the duration of hiccups, which are 1) hiccup bouts, 2) persistent hiccups, and 3) intractable hiccups [5]. Sex differences in the frequency of common hiccups have not been detected in healthy subjects. Although the onset of persistent or intractable hiccups shows a male dominance, Lee et al. reported that hiccups of a non-CNS origin are more common in men. A male dominance was not found for hiccups of a CNS origin [6]. A large number of studies have examined the causes of hiccups, which have been classified into psychogenic, organic, and idiopathic [7].

Previous studies investigated hiccups-related drugs [8–12]. Fauzia Nausheen et al. described the following about the neurotransmitters related to hiccups in their review [13]. The reflex arc is potentially mediated by central neurotransmitters (GABA, dopamine, and serotonin) and peripheral neurotransmitters (epinephrine, norepinephrine, acetylcholine, and histamine). In drugs related to GABA, propofol, benzodiazepine, and barbiturates have been identified as drugs that induce hiccups. On the other hand, valproic acid, baclofen, and gabapentin are used in the treatment of hiccups. In drugs related to dopamine, dopamine agonists, aripiprazole, and levodopa have been identified as drugs that induce hiccups. On the other hand, a number of studies have stated that chlorpromazine is effective against hiccups. Although there is no evidence for drugs related to serotonin inducing hiccups, olanzapine and risperidone have been used in the treatment of hiccups. In drugs related to histamine, omeprazole was reported to exert inhibitory effects on hiccups. M Steger et al. proposed that proton pump inhibitors (PPIs) are the first choice of medical treatment for hiccups in their systemic review [14]. Methylphenidate and metoclopramide were identified as drugs that control hiccups by influencing epinephrine and acetylcholine, respectively.

Chemotherapy-induced hiccups were recently reported. A relationship was found between anti-cancer drugs and hiccups, and a large number of studies have described hiccups caused by cisplatin [11, 12]. Furthermore, antiemetic medications, which are often combined with anti-cancer drugs, have attracted attention as a risk factor for hiccups. Lee et al. succeeded in decreasing the induction of hiccups by changing patient medication from the antiemetic drug dexamethasone to methylprednisolone in a patient who developed hiccups as a result of a dexamethasone-containing chemotherapy regime [15]. They concluded that dexamethasone-induced hiccups may be controlled by steroid rotation. The clinical features and mechanisms responsible for the pathogenesis of hiccups have been partially elucidated. However, drug-induced hiccups have not yet been cyclopedically examined and a unified view on the factors involved in hiccups does not exist. Therefore, we used the Japanese Adverse Drug Event Report (JADER) database to analyze the factors associated with hiccups in terms of medications and patient characteristics. We examined the influence of dexamethasone on hiccups by analyzing patients who received chemotherapy with dexamethasone. JADER is a spontaneous reporting database that was opened to the public by the Pharmaceuticals and Medical Devices Agency (PMDA). When we use spontaneous reporting databases, various biases, such as a notoriety bias, co-prescription bias, and competition bias, may affect study outcomes [16–20].

## Materials and methods

### Database information

Since April 2004, PMDA has reported cases of adverse events associated with medications in JADER [20]. JADER is a large-scale database that contains the adverse effects of medications and patient information in Japan. We downloaded JADER from the PMDA homepage and performed our analysis. We used 378,533 cases of data from JADER between April 1, 2004 and January 20, 2016 [20].

### Production of a data analysis table

**Fig 1:**
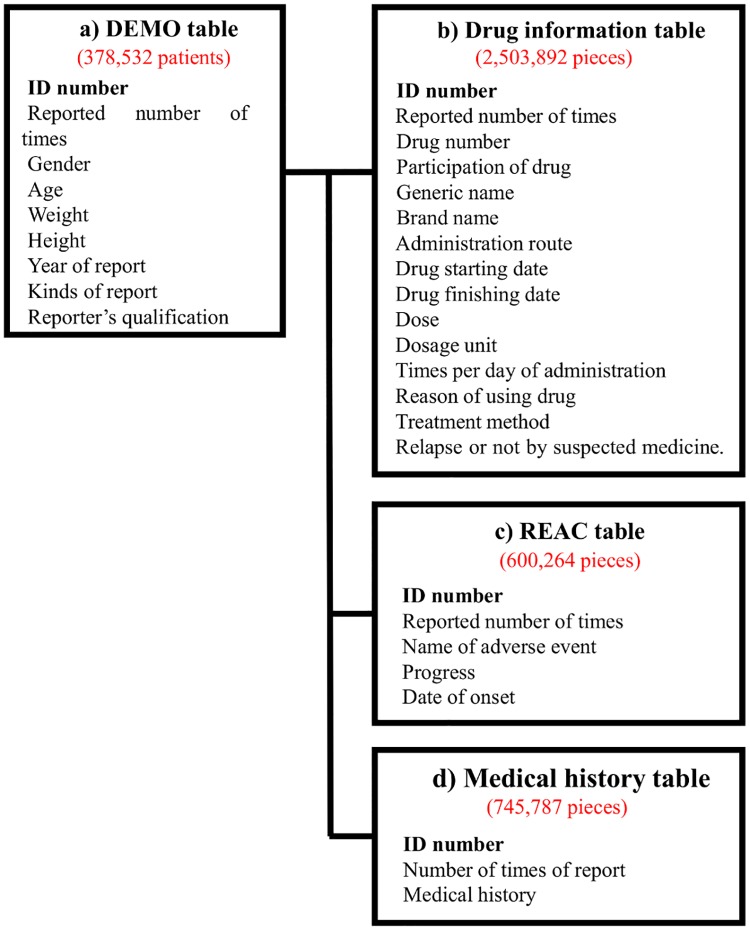
Four information tables included in JADER. Four tables were included in the JADER database. The red number shows the number of reports obtained between April 2004 and January 20, 2016.

We analyzed all data and investigated patient backgrounds and drugs that cause hiccups. JADER consists of four tables: (a) a DEMO table, (b) drug information table, (c) REAC table, and (d) medical history table. Fig 1 shows the items included in each table and the number of reports obtained between April 2004 and January 2016 [20]. We used three tables (a, b, and c) for analyses in this study.

**Fig 2:**
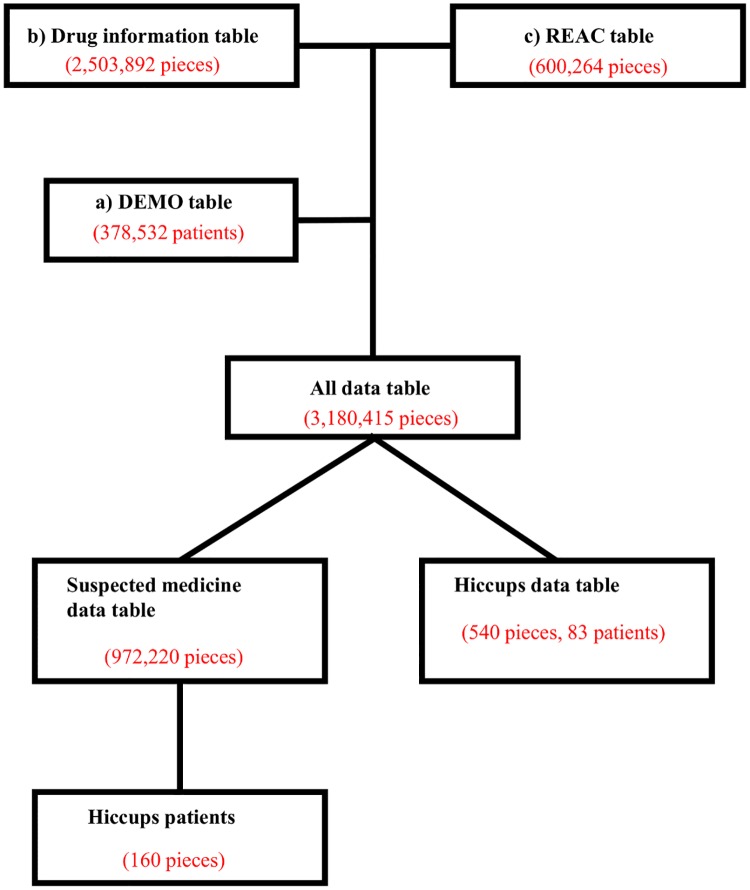
Flowchart for the construction of data analysis tables. We removed duplicated data from the drug information table (b) and REAC table (c) [21]. The DEMO table (a) was combined with the REAC table (c) and drug information table (b) using the ID number for each table. This table was called the “all data table.” In each patient, the causes of medication-related adverse events were classified into three categories: “suspected medicine,” “concomitant medicine,” and “interaction”. We only extracted the cases that were classified as “suspected medicine” into the “suspected medicine data table.” We then extracted cases with the adverse effect of “hiccups” from this table. In the same manner, we extracted cases of hiccups from all data tables. This information was shown in the “Hiccups data table.”.

We removed duplicated data from the drug information and REAC tables [21]. The DEMO table was then connected to the REAC and drug information tables using the ID number of each adverse effect case. This table was called the “all data table.” Fig 2 shows a flowchart for the construction of data analysis tables.

In each case, the contribution for the adverse events of medications given was classified into three categories: “suspected medicine,” “concomitant medicine,” and “interaction.” We only extracted cases that were classified as “suspected medicine.” This information was shown in the “suspected medicine data table.” A “suspected medicine” is defined as a pharmaceutical product with which an adverse effect is suspected to be associated. A “concomitant medicine” is defined as other pharmaceutical products that are used at the time of the expression of an adverse effect. When the reporter suspects an interaction, he/she reports it as an “interaction.”

### Relationship between patient information and hiccups

We performed Fisher’s exact test and calculated *P* values according to gender for the hiccups and non-hiccups groups in the suspected medicine data table, which included 972,220 cases. Each item had missing values, and the data table was analyzed only with the data that did not include the missing values. We analyzed age, height, and weight, conducted a *t*-test, and calculated *P* values. Age, height, and weight data in JADER are reported in the form of age in decades, height in centimeter-denominated ranges, and weight in kilogram-denominated ranges. We treated these data as absolute numbers and analyzed them as continuous variables.

### Relationship between suspected medicines and hiccups

**Fig 3:**
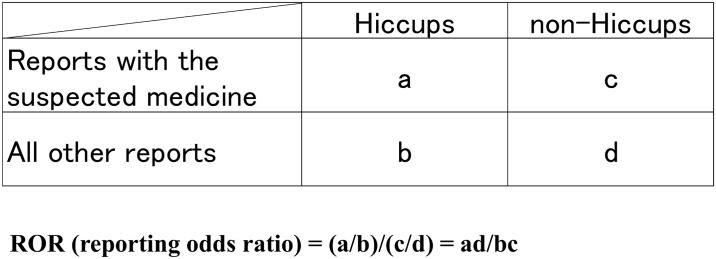
Cross-tabulation and calculation formula of the ROR of hiccups. The cross-tabulation is structured with reports for the suspected drug, all other reports, reports with hiccups, and reports without hiccups (a–d indicate the number of cases). The reporting odds ratio (ROR) was calculated as shown.

**Fig 4:**
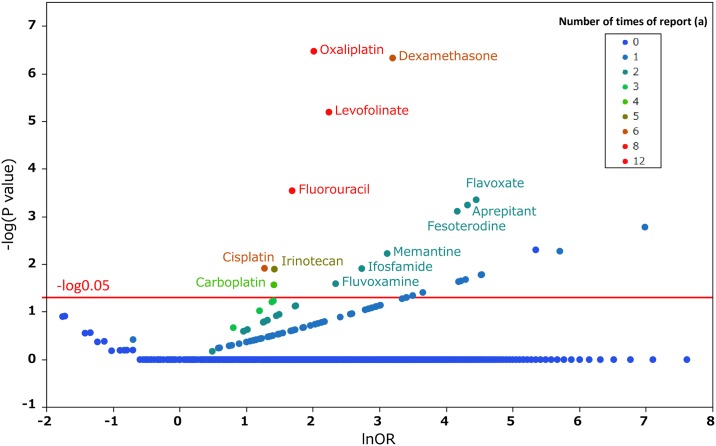
Medicines associated with hiccups. This figure shows the relationship between adverse effects such as hiccups and the medicine suspected to have caused them. The X-axis represents the reporting odds ratio (ROR) in the lnOR scale, and the Y-axis represents the *P* value in a–log_10_*P* scale. The red line shows the baseline of *P* = 0.05. Plot colors show differences in the number of patients who reported hiccups. The odds ratios were calculated through cross tabulation, as shown in Fig 3. Blue-to-red-color points indicate the number of times the adverse effect was reported (from 0 to 12).

We compiled a cross-tabulation table based on two classifications: the presence or absence of hiccups and the presence or absence of the suspected medicine. Therefore, we calculated the *P* value in Fisher’s exact test and the reporting odds ratio (ROR) (Fig 3). The ROR is the ratio of *one reporting specific adverse effect versus all other adverse effects for a given drug* to *this reporting odds for all other drugs present in the database*. In addition, it was frequently used with spontaneous reporting database as a measure of the relative risk for drug-associated adverse events. A signal is considered when the lower limit of the 95% confidence interval (CI) of the ROR is greater than one. Furthermore, we compiled a scatter-plot (volcano plot) (Fig 4) [22]. A volcano plot was constructed by plotting the negative log of the *P*-value (-log_10_*P*) from Fisher’s exact test on the y-axis and the x-axis is the log of the ROR (lnOR). Therefore, we identified medicines that influenced the onset of hiccups. S1 Table provides *P*-values and ROR values.

### Multivariate analysis

We conducted a multiple logistic regression analysis. The data that we used for the multivariate analysis included 436,190 cases, except data with missing values in the patient backgrounds of 972,220 cases. The objective variable was set to hiccups. The explanatory variables were set to patient characteristics (sex, weight, height, and age) and suspected medicines that significantly differed in the variable analysis. In this analysis, we used suspected medicines for which there were more than three reports of hiccups.

### Relationship between dexamethasone and hiccups

In order to investigate the influence of the different administration routes of dexamethasone on hiccups, we compiled a cross-tabulation table based on classifications: the presence or absence of hiccups and the administration route of choice. We calculated the *P* value in Fisher’s exact test. We also extracted data on the dexamethasone-use and anti-cancer drug-use groups in hiccups cases and investigated the involvement of suspected medicine, concomitant medicine, and interaction in each group.

### Statistical analysis

Means (± standard deviation) were calculated for all continuous variables. A *P*-value of <0.05 was considered significant. We estimated the internal correlation using the pairwise method. When the square of Spearman’s rank-order correlation coefficient [ρ^2^] was greater than 0.9, we concluded that there was an internal correlation. When there was no internal correlation, we treated these items as independent factors. All analyses were performed with JMP^®^Pro12 (SAS Institute Inc. NC, U.S.A.).

## Results

### Presentation of data

The drug information table included 2,503,892 cases, the REAC table included 600,264 cases, and the list of cases included 378,532 cases. The total number, as shown in the “all data table”, was 3,180,410. The “suspected medicine data table,” which only extracted suspected medications from the all data tables, totaled 972,220 cases.

### Hiccups and patient background

In the “suspected medicine data table,” hiccups were reported in 160 cases. Table 1 shows patient backgrounds in the non-hiccup and hiccup groups. More than 95% of patients in the hiccup group were men. The means (± standard deviation) of age, height, and weight were 53.6 ± 20.4 years, 151.6 ± 19.1 cm, and 49.8 ± 15.8 kg, respectively, in the non-hiccup group. These means (± standard deviation) in the hiccup group were 57.7 ± 14.9 years, 163.3 ± 7.9 cm, and 58.7 ± 9.9 kg, respectively. The univariate analysis showed significant differences in gender, age, height, and weight between the hiccups and non-hiccups group. Table 1 shows the *P* values of each item. In the gender analysis, we calculated the *P* value using Fisher’s exact test. In the analysis of age, height, and weight, we calculated the *P* value using a *t*-test.

**Table 1:** Patient backgrounds.

Patients	Non-hiccups	Hiccups	*P* value
**Gender (male/female)**^#^	486953/466568(953,521)	150/6(156)	<0.001^###^
Age*	53.6 ± 20.4(929,299)	57.7 ± 14.9(156)	<0.001***
Height (cm)*	151.6 ± 19.1(458,065)	163.3 ± 7.9 (91)	<0.001***
Weight (kg)*	49.8 ± 15.8(537,933)	58.7 ± 9.9(91)	<0.001***

Each item included missing values. Analyses were performed using data without missing values. The numbers in the parentheses were the numbers of cases used in the analyses.

^#^: Hiccups and non-hiccups groups were compared using Fisher's exact test

*: Hiccups and non-hiccups groups were compared using a *t*-test.

***: ^###^: *P* < 0.001

**Fig 5:**
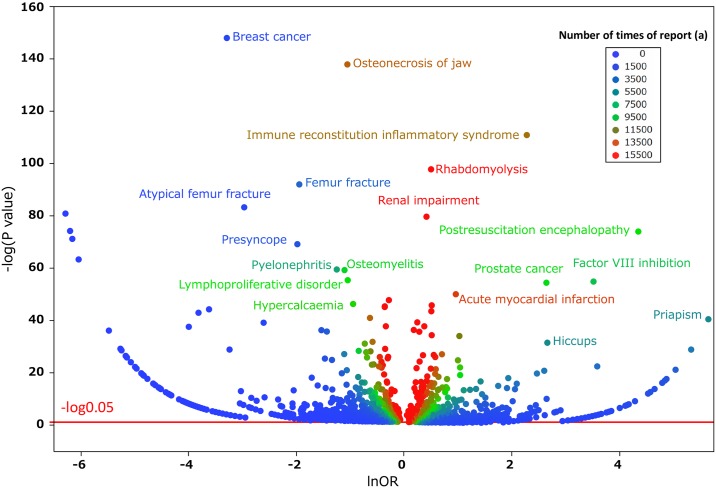
Adverse effects associated with gender. This figure shows the relationship between adverse effects and gender. The X-axis represents the reporting odds ratio (ROR) in the lnOR scale, and the Y-axis represents the *P* value in a–log_10_*P* scale. The red line shows the baseline of *P* = 0.05. Plot colors show differences in the number of patients who reported adverse effects. Blue-to-red-color points indicate the number of times the adverse effect was reported (from 0 to 15,500). ROR = (number of reports with the adverse effect in men) (number of reports with all other adverse effects in women)/(number of reports with the adverse effect in women) (number of reports with all other adverse effects in men).

In order to investigate the effects of published years on adverse effects, we calculated the reported frequency of hiccups in each year. We also calculated all reporting ratios in each year as well as Spearman’s rank correlation (ρ) between hiccups and the reporting year (ρ = −0.4384, *P* = 0.1775). The results obtained showed that there were no significant effects of the reporting year on hiccups. We also compiled a scatter-plot (volcano plot) on adverse effects and gender (Fig 5).

### Hiccup-inducing medications

**Fig 6:**
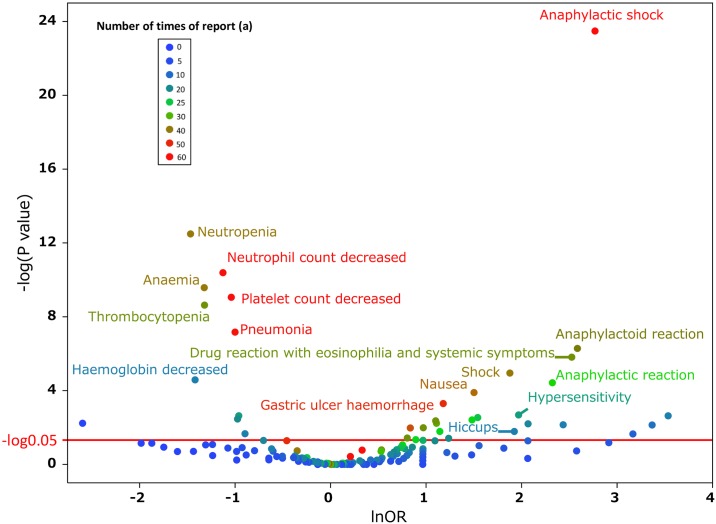
Adverse effects associated with the intravenous route. This figure shows the relationship between adverse effects of dexamethasone and the intravenous route. The X-axis represents the reporting odds ratio (ROR) in the lnOR scale, and the Y-axis represents the *P* value in a–log_10_*P* scale. The red line shows the baseline of *P* = 0.05. Plot colors show differences in the number of patients who reported adverse effects. Blue-to-red-color points indicate the number of times the adverse effect was reported (from 0 to 60). ROR = (number of reports with the adverse effect in intravenous route) (number of reports with all other adverse effects in other route)/(number of reports with the adverse effect in other route) (number of reports with all other adverse effects in intravenous route).

We cyclopedically produced a scatter plot on the relationship between the ROR and significant differences in order to examine medicines suspected of causing hiccups and other drugs (Fig 4). We used a volcano plot, which is frequently employed for the visual representation and understanding of genetic expression trends in the field of bioinformatics (Figs 4–6). The X-axis represents the log of the report odds ratio (lnOR). The positive direction of the X-axis indicates that there were more hiccup adverse effects reported than other adverse effects. The Y-axis represents the negative log of the *P*-value (-log_10_*P*) of Fisher’s exact test. The positive Y-axis direction represents a strong significant difference. In other words, for medications drawn in the upper right side, the ROR and significant difference were large. Table 2 shows the *P* value and ROR of the drugs for which the *P* value obtained using Fisher’s exact test was significant in more than three reports. Among the seven selected medications, dexamethasone and fluorouracil were the only ones available via multiple administration routes. Furthermore, the name of the medication registered with JADER may vary according to the administration route. We treated medicines with generic names associated with dexamethasone and fluorouracil to accurately evaluate the drug’s effects on hiccups.

**Table 2:** Results of Fisher’s exact test based on the presence or absence of hiccups with each medicine. N = 972,220.

Medicine	Reported number of times	Odds ratio	CI (95%)	P value
**Dexamethasone**	6	22.6	9.97–51.09	<0.0001***
**Levofolinate**	8	8.8	4.34–18.00	<0.0001***
**Oxaliplatin**	12	7.2	4.00–12.95	<0.0001***
**Fluorouracil**	8	5.1	2.50–10.36	0.0003**
**Irinotecan**	5	3.8	1.55–9.18	0.0123*
**Carboplatin**	4	3.7	1.35–9.86	0.0264*
**Cisplatin**	5	3.3	1.46–7.45	0.0118*

“Reported number of times” shows the number of cases that reported a suspected medicine for hiccups

*: p < 0.05,

**: p < 0.001,

***: p < 0.0001

### Multiple logistic regression analysis

Table 3 shows the results of a multiple logistic regression analysis performed on the seven medicines shown in Table 2 according to patient background. The multiple logistic regression analysis identified a male gender, dexamethasone, levofolinate, fluorouracil, oxaliplatin, carboplatin, irinotecan, and greater height as important risk factors for hiccups. Cisplatin was associated with the induction of hiccups, but was not an independent factor. Furthermore, age and weight showed a correlation in the univariate analysis, but were not independent risk factors. There were no internal correlations in this analysis. In other words, all the items included in the multivariate analysis were independent, and we treated them as variables that do not affect each other. For example, there was no strong correlation between gender and height; therefore, they were treated as variables that do not influence each other.

**Table 3:** Results of a multiple logistic regression analysis using medication variables and patient background variables exhibiting significance. N = 436,190.

Risk factor	*P* value	Odds ratio	CI (95%)
**Male gender**	<0.0001	32.62*	6.79–586.12
**Dexamethasone**	<0.0001	31.85*	9.60–78.32
**Levofolinate**	0.0001	8.89*	3.41–19.15
**Carboplatin**	0.0026	7.65*	2.31–18.79
**Fluorouracil**	0.0002	6.46*	2.67–13.35
**Oxaliplatin**	0.0002	5.59*	2.45–11.13
**Irinotecan**	0.0294	3.89*	1.17–9.53
**Cisplatin**	0.0508	3.30	0.99–8.09
**Height**	0.0012	1.05*	1.02–1.09
**Weight**	0.1954	1.01	0.99–1.03
**Age**	0.2478	1.01	0.99–1.03

*: indicated a significant odds ratio

### Relationship between dexamethasone and hiccups

Table 4 shows the number of patients with the use of dexamethasone and hiccups. An intravenous injection of dexamethasone was more common in the hiccups group than in the non-hiccups group. No significant differences were observed for any other administration routes, including oral administration. We also compiled a scatter-plot (volcano plot) on the adverse effects of dexamethasone and the intravenous route of drug administration (Fig 6).

**Table 4:** Fisher’s exact test results according to the presence and absence of hiccups and the route of dexamethasone administration.

Route	Non-hiccups	Hiccups	*P* value
**Total**	5970	8	-
**Intravenous**	1641	6	<0.0001
**Oral**	3379	2	0.108
**Others**	950	0	1

The total number of cases of hiccups reported in the “all data table” was 540. We removed a duplication case involving a patient with the same ID number and estimated the number of cases of hiccups. As a result, 83 cases of hiccups were reported. Of these cases, 17 patients (20%) were administered dexamethasone that was classified as the suspected medicines, the concomitant medicines, and the interaction medicines. Among the patients using dexamethasone, 16 (94%) were also using an anti-cancer drug. Sixty-six patients (80%) with hiccups were dexamethasone non-users, and twenty-four of these (36%) also used an anti-cancer drug (Table 5).

**Table 5:** Use of anti-cancer drugs in hiccup patients based on dexamethasone use.

Hiccups (83 patients)	Dexamethasone	No dexamethasone
**Anti-cancer drugs**	16	24
**No anti-cancer drugs**	1	42

## Discussion

### Database

Among the 378,532 JADER adverse effect cases, there were 160 cases of hiccups with suspected medications. In a comparison with other adverse effect reports, this was a relatively small number of cases. We suggest that patients do not consider hiccups to be an adverse effect unless they are intractable. Therefore, we infer that reports of hiccups in this database are mostly refractory hiccups. By only analyzing suspected medicines reported in JADER, we may have limited the adequate identification of the causal drug. Therefore, we examined all data including the suspected medicine, concomitant medicine, and interaction for dexamethasone and anti-cancer drugs.

### Hiccups and gender

The incidence of persistent or intractable hiccups is significantly higher in men. Lee et al. showed a male dominance in their meta-analysis. Our results supported these findings (OR: 32.6, 95% CI: 6.8–585.1). Kitraki et al. reported the specificity of steroid receptors in the brain and pituitary gland in the rat based on gender [23]. This may have influenced the male dominance in dexamethasone-induced hiccups. Furthermore, we compiled a scatter-plot (volcano plot) on adverse effects and gender (Fig 5) [22]. We found that adverse effects varied according to gender. Hiccups showed a strong signal in the area of male intentionality. Other adverse effects plotted in this area were adverse effects in men. These results reconfirmed a male predominance for adverse effects using the JADER database.

### Hiccups and patient background

We conducted an investigation into the relationship between hiccups and patient characteristics (age, height, and weight) included in JADER. The results obtained showed that an advanced age, greater height, and greater weight were all significant factors positively influencing the onset of hiccups. We also conducted a multiple logistic regression analysis to extract independent factors. Only a greater height was identified as an independent risk factor for hiccups (OR 1.05, 95% CI: 1.02–1.09). To the best of our knowledge, the relationship between hiccups and physical features has not yet been examined. This study is the first to show a relationship between height, weight, and hiccups. Therefore, we may be able to predict the onset of hiccups from medicines taken and patient characteristics. These results may assist in the choice of appropriate therapeutic medicines and preventive medicines for patients with an elevated risk of hiccups. Further clinical trials are required.

### Hiccup-inducing medicines

The results of the present study showed that chemotherapy-related drugs induced hiccups. The multivariable analysis indicated that anti-cancer drugs and dexamethasone are independent risk factors for hiccups.

Drug-induced hiccups are regarded as peripheral hiccups [6]. The induction of hiccups by anti-cancer drugs has been attributed to peripheral neuropathy and direct irritation of the organ. Among the medications in this examination, platinum drugs including oxaliplatin and cisplatin are known to cause peripheral neuropathy. The mechanism responsible for the induction of hiccups by cisplatin is the stimulation of the vagus nerve by the release of serotonin as a result of the stimulation of enterochromaffin cells. Furthermore, the developmental mechanism underlying anti-cancer drug-induced peripheral neuropathy may be nervous demyelination. The hiccup is a reflex arc that passes through afferent, efferent, and central pathways of the nervous system. It is important to consider the effects of drugs causing peripheral neuropathy; however, a relationship between peripheral neuropathy and hiccups has not yet been proven. In patients receiving chemotherapy, it is clinically important to identify medicines that induce hiccups and elucidate the underlying mechanisms because hiccups reduce the quality of life of patients. In the present study, the medications that were closely related to the induction of hiccups are shown. These results may be helpful for selecting medications in clinical settings and may also be beneficial for patients undergoing chemotherapies.

### Dexamethasone-induced hiccups

Our investigation on dexamethasone administration routes and hiccups revealed a relationship between the intravenous route and induction of hiccups. Dexamethasone is often used as an antiemetic in chemotherapy [24]. We were unable to show a clear relationship between dexamethasone doses and the induction of hiccups in the present study. The following bias is regarded as influencing the relationship between adverse events and drug administration routes. Patients intravenously receiving medications are more frequently observed by physicians than patients receiving oral medications, with the former group being more likely to report adverse effects than the latter. In order to consider the bias described above, we compiled a scatter-plot (volcano plot) on the adverse effects of dexamethasone and drug administration routes (Fig 6) [22]. Many hiccups were confirmed as an adverse effect by the intravenous route. However, its signal was not as strong as those of the other adverse effect reports. There may have been too few adverse effect reports on hiccups to analyze the relationship between hiccups and admission routes.

We then examined the effects of dexamethasone combined with other medicines. The suspected medicine, concomitant medicine, and interaction of the drug for the adverse event were selected by reporters in JADER. We often use medication data of the suspected medicine for an analysis in JADER. However, there is a large report bias associated with using only the data of the suspected medicine. Therefore, we conducted an examination of all data for the suspected medicine, concomitant medicine, and interaction. As a result, most of the reported cases of dexamethasone use in our study were in combination with an anti-cancer drug (16 out of 17). Among dexamethasone users, only one case did not use an anti-cancer drug; this was a case of an intravenous injection of medicine for asthma. In this case, there were no medicines that induced hiccups other than dexamethasone. Therefore, dexamethasone use alone appears to induce hiccups. This result suggests that dexamethasone is an important drug associated with the induction of hiccups.

High-dose dexamethasone passes the blood-brain barrier and activates steroid receptors in the hypothalamic hippocampus [25]. Therefore, the efferent pathway of the hiccup reflex arc is stimulated. Lee et al. reported that the incidence of hiccups decreased in a patient after changing from dexamethasone to methyl prednisolone during chemotherapy. However, they reported that 15% of patients still had hiccups even after they changed from dexamethasone to other medications [15]. Therefore, the induction of hiccups during chemotherapy in not solely attributed to dexamethasone use.

In our examination, there were 66 cases of hiccups in patients who did not use dexamethasone. Of those, 23 patients were using anti-cancer drugs. This supports the results of the multivariable analysis showing that the use of anti-cancer drugs is one of the independent risk factors influencing the onset of hiccups. Anti-cancer agents, except for platinum anti-cancer drugs, were extracted as suspected hiccup-inducing medicines in our study. Further studies are needed in order to elucidate the mechanisms responsible for anti-cancer drug-induced hiccups.

### Limitations of this study

Since JADER is based on a self-reporting system, these cases are limited. Mild adverse effects are only occasionally reported, with severe cases being reported more often. This a known report bias, which is characteristic of a self-report database [26]. This database was problematic given that the association between medicines and adverse events was not clear [27]. If several drugs are concomitantly administered to a single patient, it is difficult to identify the causes of an adverse drug reaction [17–20]. PMDA verifies details on fatal cases due to adverse events and examines the relationship between medicines and adverse events. However, other cases are reported based on judgments by the reporters. The cases in JADER included not only true, but also suspected adverse effects.

The results of the present study suggest that hiccups are more common in men than in women and in those who are tall than in those who are short. However, we need to introduce assumptions that the frequency of reporting adverse effects to JADER does not depend on either the sex or height of patients. Hiccups are more common among those intravenously administered dexamethasone, suggesting that the method of administration is independent of reporting. Neither of these assumptions may be plausible. Patients intravenously administered medicines are more likely to be seen by a physician and may be more likely to report an adverse effect. Furthermore, as a result of co-medications, reporting may be more frequent than in patients taking only one medication because of the differentiation of causality of physicians; this may result in bias in the multiple logistic regression analysis.

## Conclusion

We searched data on medications and patient characteristics associated with hiccups using the large JADER database. Our results suggest that not only medications, but also patient backgrounds are associated with hiccups. By using large-scale adverse effects report data, we were able to extract medications that induced the rare adverse effect of hiccups. The results of the present study will assist practitioners in the selection of appropriate therapeutic medicine, potentially avoid this adverse effect, and improve patient quality of life.

## Supporting information

S1 Table Reporting odds ratios and P values of Fisher’s exact test between the pathogenesis of hiccups and drugs. Drugs with the highest *P* values (*P* = 1) were omitted from this table.
(DOCX)
