# Supplementary material for: Analysis of factors associated with hiccups based on the Japanese Adverse Drug Event Report database
Source: PLoS One. 2017 Feb 14;12(2):e0172057. doi: 10.1371/journal.pone.0172057 (PMC5308855; doi:10.1371/journal.pone.0172057)
Supplement: S1 Table — Drugs with the highest P values (P = 1) were omitted from this table. (DOCX) [file pone.0172057.s001.docx]

Table S1. Reporting odds ratios and P values of exact test between pathogenesis of hiccups and drugs

Drugs with the highest P value (P = 1) were omitted in this table.
